# Combined Type Mediastinitis After Thyroidectomy Managed by Negative Pressure Wound Therapy With Instillation: A Case Report

**Published:** 2019-02-26

**Authors:** Daiki Kitano, Yoshitaka Kitamura, Koichiro Yonezawa, Wataru Nishio, Shigemichi Iwae, Minoru Nakahara, Shunsuke Sakakibara

**Affiliations:** ^a^Departments of Plastic Surgery; ^b^Thoracic Surgery; ^c^Head and Neck Surgery, Hyogo Cancer Center, Akashi, Hyogo, Japan; ^d^Department of Plastic Surgery, Kobe University Graduate School of Medicine, Kobe, Hyogo, Japan

**Keywords:** descending necrotizing mediastinitis, poststernotomy mediastinitis, thyroidectomy, negative pressure wound therapy, NPWTi

## DESCRIPTION

A 52-year-old woman with a history of dyslipidemia, panic disorder, asthma, a 32-pack-year smoking habit, and obesity (body mass index [BMI] = 30.0 kg/m^2^) underwent subtotal thyroidectomy with cervical and mediastinal lymphadenectomy through a median sternotomy due to papillary thyroid cancer. Median sternotomy was performed during superior and anterior mediastinal lymph node dissection. The sternum was rewired, and a continuous drainage tube was placed at the anterior mediastinum.

On the second postoperative day, extubation was attempted; however, she developed dyspnea. Considering the risk of descending necrotizing mediastinitis (DNM) after tracheostomy, we performed cricothyrotomy, which required a smaller skin incision than tracheostomy. However, spiking fever was noted on eighth postoperative day and the value of C-reactive protein (CRP) was mildly elevated at 6.97 mg/dL. The cervical and chest wounds were erythematous and purulent discharge exited from the drain hole in the epigastric region on the 20th postoperative day. Computed tomographic (CT) scan showed increased CT value of subcutaneous tissue, fluid collection, and free air in the cervical and anterior mediastina. The edge of the rewired sternum was uneven and wire cutting of the bone was noted.

Under general anesthesia, the mediastinal wound was reopened and a large amount of yellow, foul-smelling pus collection was confirmed. The sternal wound culture yielded *Streptococcus constellatus/milleri*, *Prevotella intermedia*, and *Actinomyces meyeri*, which respectively consist of normal oropharyngeal flora. The mediastinal wound communicated with the tracheostomy stoma, and this was thought to be the route of infection. To prevent further infection from the cervical area, the subcutaneous tissue below the stoma was sutured after adequate mediastinal debridement and curettage of the tracheostomy stoma.

For the management of the sternal wound after debridement, we used a negative pressure wound therapy (NPWT) with instillation (NPWTi) device. Trex Siliconized Gauze (Fuji Systems Corp, Tokyo, Japan) was placed on the mediastinal tissue as a contact layer for prevention of critical bleeding from the heart and great vessels. After BLAKE Silicone Drains Hubless (Ethicon) was placed on the Trex Gauze, Granufoam (Kinetic Concepts Inc, San Antonio, Tex) was packed into the sternal wound. A normal saline solution was instilled at 100 mL/h into the mediastinal cavity through the tube. By utilizing the ActiV.A.C. Therapy Unit (Kinetic Concepts Inc), continuous negative pressure of 100 mm Hg was applied to the wound via the foam. The foam dressing was changed twice a week under general anesthesia, and additional debridement was conducted at the same time, as needed. Piperacillin/tazobactam 4.5 g every 6 hours and vancomycin 1 g every 12 hours were administered intravenously.

Three days after NPWTi was started, the subcutaneous connection between the tracheostomy stoma and the sternal wound was completely separated with adhesion of granulation tissues growing from the supraclavicular region. Because CRP values and cultures from the mediastinum were negative, the mediastinal wound was reconstructed with bilateral advanced pectoral major muscle flap on the 15th postdebridement day. Her postoperative course was satisfactory, and she was discharged 29 days after mediastinal reconstruction.

## QUESTIONS

1. What is poststernotomy mediastinitis (PSM)?
2. What is descending necrotizing mediastinitis?
3. How is combined type mediastinitis managed?
4. Why NPWTi is effective?

## DISCUSSION

Poststernotomy mediastinitis is defined as a mediastinitis associated with sternal osteomyelitis after median sternotomy.^1^ The incidence of PSM is 1% to 3% in open heart surgery, and its mortality rate is reported to vary between 10% and 25%.^2^ Wire cutting through the sternum is found to be a cause of sternal dehiscence.^3^ Obesity with a BMI over 30.0 kg/m^2^, as in our case, has been clarified to be an important risk factor of PSM because it precipitates wire cutting and subsequent sternal dehiscence.^4^

Descending necrotizing mediastinitis is a serious infection with a 10% to 40% rate of death, in which odontogenic, pharyngeal, or cervical infection spreads by the subcutaneous tissue and cervical fascia to the mediastinum.^5^ In 1983, Estrera et al^6^ described the diagnostic criteria as follows: (*a*) clinical manifestations of oropharyngeal or severe cervical infection, (*b*) demonstration of characteristics of mediastinitis on radiological imaging study, (*c*) documentation of mediastinal infection at operation or postmortem examination, and (*d*) establishment of relationship between oropharyngeal infection and mediastinitis. Because our case fulfilled all these 4 criteria, we made a diagnosis of DNM.

Because our case met the diagnostic criteria of both PSM and DNM, we established the concept of “combined type mediastinitis.” Taking into account the high mortality rate of PSM and DNM, we needed to ensure appropriate treatment of combined type mediastinitis. We sealed the subcutaneous connection between the tracheostomy stoma and the mediastinum by suturing and negative pressure induced by NPWTi after adequate debridement of the cervix and the mediastinum. This enabled us to reduce the treatment strategy for combined type mediastinitis to the treatment equivalent for PSM. In the treatment of PSM, it has been certified that aggressive debridement in addition to antibiotic therapy improved the prognosis.^7^ When sternal osteomyelitis is presented as a comorbidity of PSM, debridement of the infected and necrotized sternum with peripheral soft tissue and, in severe cases, the costal cartilage is recommended.^8^ In our case, sufficient debridement of infected tissue including subtotal resection of the necrotic sternum was the critical element of the treatment.

In recent years, some researchers have proposed NPWT as the first-line therapy for PSM because of its excellent outcomes.^9^ NPWTi is the device that combines negative pressure and instillation of solutions for the management of intractable ulcers. Specifically, for the management of an infected wound after appropriate surgical debridement, it was shown that NPWTi provided better clinical outcomes than those achieved by NPWT alone.^10^ NPWTi was also effective in the management of combined type mediastinitis after thyroidectomy.

## SUMMARY

We report a case of “combined type mediastinitis” after thyroidectomy with median sternotomy. The subcutaneous communication between the mediastinal wound and the tracheal stoma was the route of infection. After careful debridement, NPWTi was crucial to successful management of combined type mediastinitis.

## Figures and Tables

**Figure 1:**
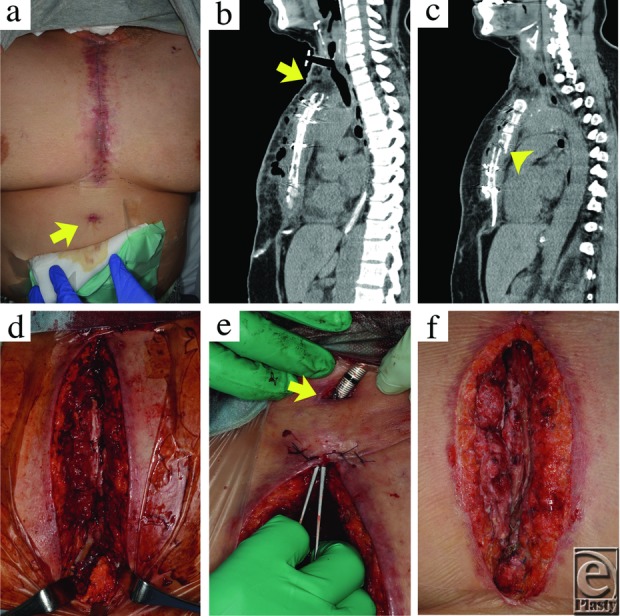
Debridement operative findings.

**Figure 2:**
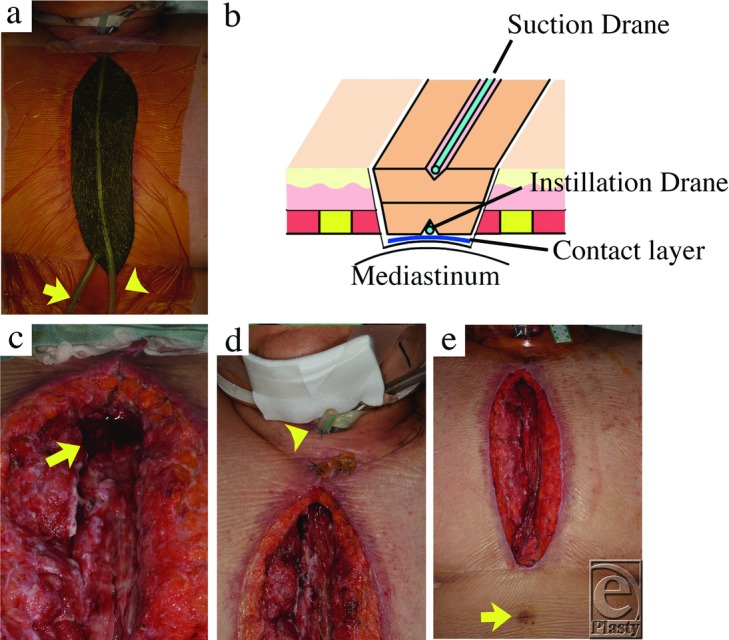
Clinical course of negative pressure wound therapy with instillation.

**Figure 3:**
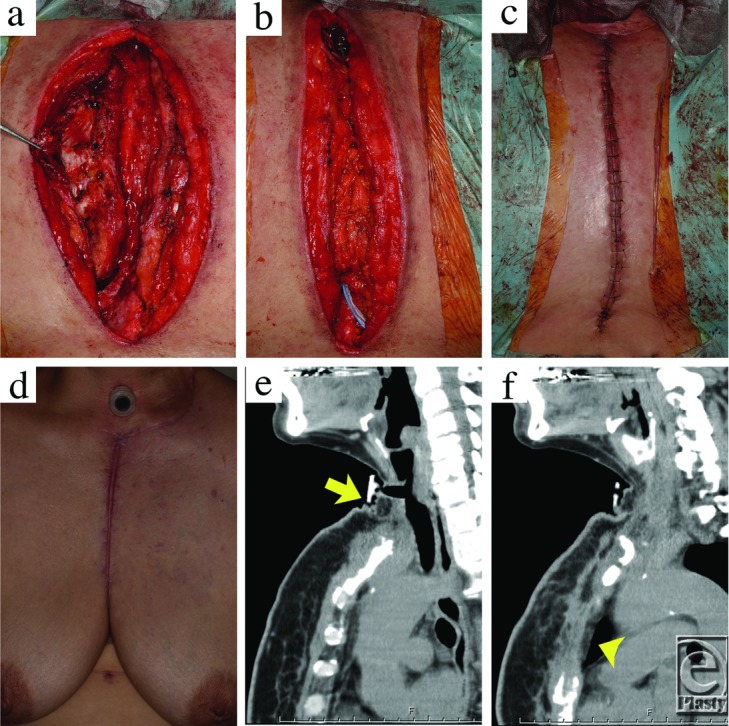
Wound closure.
